# Bidirectional Mendelian Randomization Analysis for Vitamin D and Thyroid Peroxidase Antibody

**DOI:** 10.1155/2022/2260388

**Published:** 2022-04-01

**Authors:** Yingchao Chen, Bing Han, Chunfang Zhu, Qin Li, Chi Chen, Hualing Zhai, Ningjian Wang, Yi Chen, Yingli Lu

**Affiliations:** Institute and Department of Endocrinology and Metabolism, Shanghai Ninth People's Hospital, Shanghai JiaoTong University School of Medicine, Shanghai, China

## Abstract

**Purpose:**

Whether Vitamin D deficiency or insufficiency is associated with thyroid autoimmunity was debated for long time. This study was still to explore the causal relationship of 25 (OH) D with a thyroid peroxidase antibody (TPOAb).

**Methods:**

The data were obtained from a cross-sectional study, SPECT-China study, which was performed in 23 sites in East China during 2014 to 2016. 10636 participants were finally included in this study. Genotyped four 25 (OH) D-related and four TPOAb-associated single nucleotide polymorphisms (SNPs) created their genetic risk scores (GRS). Bidirectional mendelian randomization (MR) analysis was used in this study.

**Results:**

25 (OH) D GRS was significantly associated with 25 (OH) D (B −0.093, 95% CI −0.111, −0.074) and TPOAb level (B 0.067, 95% CI 0.002 to 0.132). TPOAb GRS was significantly associated with TPOAb concentration (B 0.345, 95% CI 0.135 to 0.556), but not 25 (OH) D (B −0.030, 95% CI −0.091 to 0.030). Using 25 (OH) D_GRS as instrumental variable in the MR analysis, a causal relationship of genetically determined 25 (OH) D with increased TPOAb concentration (B −0.720, 95% CI −1.429 to −0.012). No relation was found between genetically instrumented TPOAb and 25 (OH) D.

**Conclusion:**

A higher VD_GRS was associated with higher risk of increased TPOAb concentration, which supports a causal association between decreased vitamin D and increased concentration of TPOAb in an eastern Chinese population.

## 1. Introduction

Thyroid dysfunction including overt and subclinical hypothyroidism in the population has considerable consequences for a number of health issues, including insulin resistance, metabolic syndrome, worse lipid profile, central adiposity, and obesity [1–4]. The most common cause of hypothyroidism is the Hashimoto thyroiditis (HT) and the basic mechanisms in the development of thyroid autoimmunity may be due to a combined TPO- and Tg-specific cytotoxic immune response [5]. It was reported that the prevalence of detectable thyroid antibodies, primarily TPOAb, comprises 10–12% of the healthy population [6–8]. Despite the prevalence and adverse outcomes of autoimmune-mediated thyroid disease, its etiology remains incompletely understood [9, 10].

Vitamin D deficiency is also a pandemic health problem in both developing and developed countries [11]. Recently, the actions of vitamin D have been shown to go beyond calcium/phosphorus homeostasis via bone formation and resorption to higher susceptibilities of immune-mediated disorders, including chronic infections and autoimmune diseases [12]. Several epidemiological studies showed lower vitamin D levels to the pathogenesis of increasing TPOAb [6, 13–17]. However, conflicting studies were also present reporting that no significant association between the serum vitamin D levels and thyroid autoimmunity [18–20]. Thus, whether low vitamin D levels truly associated with AITD, whether the association is causal, and if so, its causal direction, is still unclear.

The Mendelian randomization (MR) approach was taken widely used for assessing causality in population studies [21], which is the main limitation of a cross-sectional study. Using the genetic variants as the instrumental variable (IV) has become a widely-used approach for causal inference [22]. In this study, if low 25 (OH) D causally induces high TPOAb, genetic variants associated with lower 25 (OH) D should be associated with higher TPOAb concentration, and vice versa. These genetic variants are inherited independent of potential confounding factors [22]. Thus, MR could avoid problems in conventional epidemiological studies such as residual confounding and reverse causation [23].

In the present study, on the basis of the large community-based sample of Chinese participants from SPECT-China study (Survey on Prevalence in East China for metabolic diseases and risk factors), we performed a bidirectional MR approach to explore the causal association between increased TPOAb levels and decreased 25 (OH) D levels. TPOAb and Vitamin D genetic risk scores (TPOAb_GRS and VD_GRS) were constructed to represent the genetic susceptibility.

## 2. Materials and Methods

### 2.1. Study Participants

The data were from the SPECT-China study (ChiCTR1900021356), which is a large cross-sectional study. Recruitment and enrollment of the study have been previously described in detail [24–26]. From 2014 to 2016, 12666 subjects who were Chinese citizens, ≥18 years old, and had lived in their current area for ≥6 months were recruited for the SPECT-China study from 23 sites in Shanghai, Zhejiang, Jiangsu, Anhui, and Jiangxi Province. Among them, genotype information was available in 10672 participants (84.3%). We excluded the participants who missed information on more than two single nucleotide polymorphism (SNP) genotypes (*n* = 20), missing the concentration of TPOAb (*n* = 14) and 25 (OH) D (*n* = 2). 10636 participants were involved in the final analysis. All participants provided written informed consent before data collection. The study protocol was approved by the Ethics Committee of Shanghai Ninth People's Hospital, Shanghai JiaoTong University School of Medicine. All procedures followed were in accordance with the ethical standards of the responsible committee on human experimentation (institutional and national) and with the Helsinki Declaration of 1975, as revised in 2008.

### 2.2. Measurements

A single assessment protocol of interview and collection of biological specimens at each site was undertaken. Blood samples of each participant were obtained from 7 : 00 Am to 10 : 00 Am after fasting for at least 8 hours. Blood samples were refrigerated immediately after phlebotomy, and after 2∼4 hours they were centrifugation and the serum was aliquoted and frozen in a central laboratory. TPOAb was measured by the chemiluminescence immunoassay (Siemens, immulite 2000, Erlangen, Germany) and the 25 (OH) D was detected using a chemiluminescence assay (Siemens ADVIA Centaur XP, Germany). Body mass index (BMI) was calculated as weight in kilograms divided by height in meters squared.

### 2.3. Genotyping, Genetic Loci Selection, and Genetic Risk Score Construction

DNA was extracted from blood white cells using a blood genomic DNA extraction kit (DP603, TIANGEN BIOTECH CO, LTD, Beijing, China) on an automated nucleic acid extraction instrument (YOSE-S32, TIANGEN BIOTECH CO, LTD, Beijing, China). Specific assays were designed using the Geneious Pro (v4.8.3) (https://www.geneious.com/). Mass determination was carried out with the JUNO and data acquisition was used Fluidigm SNP Genotyping Analysis v4.1.3 software (Fluidigm Corporation, South San Francisco, California, USA). Call rates of all SNPs were higher than 98% [27, 28].

We selected 4 SNPs involved in susceptibility of TPOAb concentration (major histocompatibility complex, class II, DP beta 1 (HLA-DPB1)- rs9277555, thyroid peroxidase (TPO)- rs11675434, arginine-glutamic acid dipeptide repeats (RERE)- rs301799, and HLA complex P5 (HCP5)- rs3094228) for our analysis based on previously published genome-wide association study on TPOAb concentration [29]. We also selected 4 vitamin D-related SNPs (vitamin D binding protein (GC)- rs2282679, cytochrome P450 family 2 subfamily *R* member 1 (CYP2R1)- rs10741657, 7-dehydrocholesterol reductase (DHCR7)- rs12785878, and cytochrome P450 family 24 subfamily A member 1 (CYP24A1)-rs6013897) were chosen on the basis of the recent genome-wide association study on 25 (OH) D [30]. They all reached a genome-wide significance level (*P* < 5 × 10^−8^) and not in linkage disequilibrium (*r*^2^ = 0).

### 2.4. Statistical Analysis

Data were analyzed by using IBM SPSS Statistics, Version 22 (IBM Corporation, Armonk, NY, USA). All analyses were two-sided. A *P* value <0.05 was considered significant. Continuous variables were expressed as the mean (±standard deviation) values, and categorical variables were presented as percentages. 25 (OH) D and TPOAb was logarithmically transformed before analysis.

The additive genetic model for each SNP (coded as 0, 1 and 2) was used to construct GRS. For the VD_GRS, we created a weighted score by multiplying each SNP by a weight based on its effect size with 25 (OH) D obtained from a large study containing Asian population [30]. For the TPOAb_GRS, the weights were from meta-analysis of Atherosclerosis Risk In Communities study (ARIC) and Study of Health in Pomerania-TREND (SHIP) [29]. The characteristics of each SNP in the VD_GRS and TPOAb_GRS are summarized in Table 1.

To examine the strength of the allele scores as instruments, the F-statistic was approximated from the proportion of variation in the respective phenotype (*R*^2^) explained by the allele score, (F − stat = (*R*^2^^*∗*^(*n* − 2))/(1 − *R*^2^)) [31].

Linear regression analyses were used to determine the association of the two GRSs and TPOAb with 25 (OH) D, and the association of the two GRSs and 25 (OH) D with TPOAb. Model 1 adjusted for age, sex. Model 2 adjusted for terms in model 1. Model 2 adjusted for terms in model 1 and waist to hip ratio.

Regarding the MR analysis, the weighted VD_GRS and weighted TPOAb_GRS were used as the instrumental variables (IV) estimators to measure the strength of the bi-directional causal relationship between 25 (OH) D and TPOAb concentration. The formal MR analyses to estimate the possible causal effect of 25 (OH) D on TPOAb (and vice versa) were conducted using the IV ratio method [22]. For the causal association of increased risk of TPOAb in relation to lower 25 (OH) D, the computational formula was *β* (VD-TPOAb) = *β* (VD_GRS-TPOAb) /*β*VD_GRS-25 (OH) D). In the opposite direction, the computational formula was *β*IV(TPOAb-VD) = *β*TPOAb_GRS-25 (OH) D/*β*(TPOAb_GRS-TPOAb). The model was the same as the above model 2, adjusting for age, sex, and waist to hip ratio. The standard error (SE) and confidence interval (CI) for the IV estimators was estimated by the delta method. The formulas are shown below:

(1) $$\begin{array}{c} {\text{SE}}_{\text{IV}}=abs\left(\beta_{IV}\right)\sqrt{{\left(\frac{{\text{SE}}_{\text{GRS\_exposure}}}{\beta_{\text{GRS\_exposure}}}\right)}^{2}+{\left(\frac{{\text{SE}}_{\text{GRS\_outcome}}}{\beta_{\text{GRS\_outcome}}}\right)}^{2}},\\ 95\%{\text{CI}}_{\text{IV}}=\beta_{\text{IV}}\pm 1.96\times {\text{SE}}_{\text{IV}}. \end{array}$$

To validate the genetic instruments, we assessed the associations between each individual SNP with 25 (OH) D and TPOAb, respectively. We also measured the potential pleotropic associations of each individual SNP and the GRSs with age, sex, BMI, and waist to hip ratio.

## 3. Results

### 3.1. Association of Four 25 (OH) D-Related SNPs with 25 (OH) D and TPOAb

**Figure 1:**
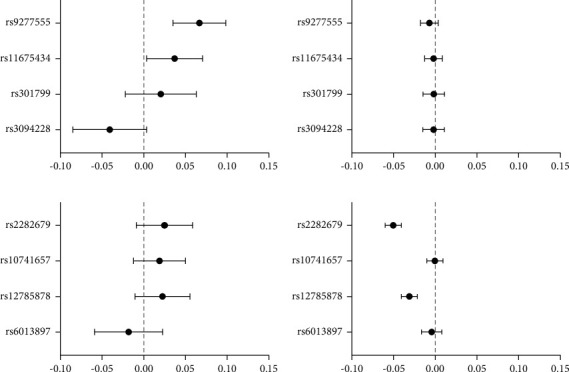
(a) Association of the four vitamin D-related SNPs with 25 (OH) D (lnVD). (b) Association of the four vitamin D-related SNPs with TPOAb concentration (lnTPOAb). (c) Association of the four TPOAb-related SNPs with TPOAb concentration (lnTPOAb). (d) Association of the four TPOAb-related SNPs with 25 (OH) D (lnVD). Data were presented as unstandardized coefficients and 95% confidence interval. The model was adjusted for age, sex, and waist to hip ratio.

The associations of each individual 25 (OH) D-related SNP with ln-TPOAb are summarized in Figures 1(a) and 1(b). In these four 25 (OH) D-related SNPs, two SNPs located at the GC (rs2282679) and DHCR7 (rs12785878) loci were significantly associated with the level of 25 (OH) D and none of them were significantly associated with the concentration of TPOAb.

### 3.2. Association of Four TPOAb-Related SNPs with 25 (OH) D and TPOAb

The associations of each individual TPOAb-related SNP with ln-25 (OH) D are summarized in Figures 1(c) and 1(d). In these four TPOAb-related SNPs, two SNPs located at the HLA-DPB1 (rs9277555) and TPO (rs11675434) loci were significantly associated with the level of TPOAb and none of them were significantly associated with the level of 25 (OH) D.

### 3.3. Pleiotropic Effects of SNPs and Weighted GRS

The association of these four 25 (OH) D-related SNPs and four TPOAb-related SNPs with major 25 (OH) D and TPOAb related confounders were calculated. Therefore, we measured the potential associations of the SNPs with age, waist to hip ratio, BMI, and sex distribution using an additive model. Unstandardized coefficients (standard error) and odds ratio (95% confidence interval) are summarized in Table 2. None of these eight SNPs had pleiotropic effects (all *P* > 0.05).

Then, the association of VD_GRS and TPOAb-GRS with major 25 (OH) D and TPOAb related confounders were calculated for further analysis (Table 3). Neither of them has significant association with these confounders (all *P* > 0.05).

### 3.4. Study Characteristics According to Weighted VD_GRS and TPOAb_GRS Tertiles

We classified study subjects into three groups according to VD_GRS tertiles (Q1: ≤0.82, Q2: 0.83–1.13, Q3: ≥1.14) and TPOAb_GRS tertiles (Q1: ≤0.136, Q2: 0.137–0.240, Q3: ≥0.241), respectively. The general characteristic of study subjects in terms of these two tertiles is shown in Table 4. As expected, with the increasing of VD_GRS, 25 (OH) D concentrations significantly decreased and with the increasing of TPOAb_GRS, TPOAb concentrations significantly increased (*P* < 0.001 and *P*=0.021). TPOAb concentrations increased significantly along with the increased VD_GRS levels (*P*=0.019). However, there is no significant difference of 25 (OH) D levels between TPOAb_GRS tertiles (*P* > 0.05).

### 3.5. Associations of VD_GRS and 25 (OH) D with TPOAb Concentration

As shown in Table 5, in this cross-sectional study, 25 (OH) D was positively associated with the level of TPOAb after adjusted for age, sex, and waist to hip ratio (*B* = 0.070, 95% CI 0.003, 0.017). Then, the association of VD_GRS and tertiles of VD_GRS with TPOAb concentration was measured. Increased VD_GRS was significantly associated with increased TPOAb levels after adjusting for age and sex (*B* = 0.066, 95% CI 0.002, 0.129) (model 1). Further adjusting for waist to hip ratio did not change the result (*B* = 0.067, 95% CI 0.002, 0.132) (model 2). The same trend was also seen in the tertiles of VD_GRS (*P* for trend = 0.027 in model 1 and 0.031 in model 2).

### 3.6. Associations of TPOAb_GRS and TPOAb with 25 (OH) D Concentration

Conversely, the association of the TPOAb_GRS and TPOAb with 25 (OH) D is shown in Table 6. Although the TPOAb level was significantly associated with the level of 25 (OH) D, increased TPOAb_GRS was not significantly associated with decreased level of 25 (OH) D in both models. The tertiles of TPOAb_GRS showed similar results (both *P* for trend >0.05).

### 3.7. 25 (OH) D and TPOAb Concentration: The Bidirectional MR Analysis

**Figure 2:**
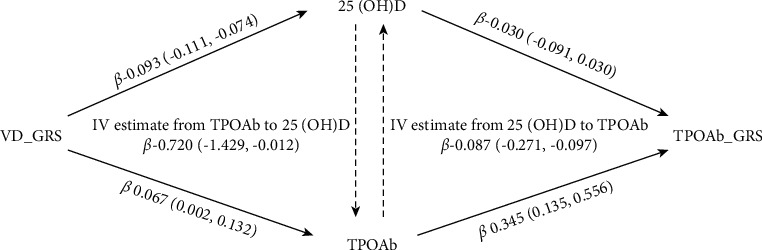
Bidirectional instrumental variable (IV) estimated association between 25 (OH) D and TPOAb (concentration) by weighted GRSs. Data were adjusted for age, sex, waist to hip ratio.

In order to elevated the strength of the allele scores as instruments, F-statistic for VD_GRS and TPOAb_GRS was calculated. For VD_GRS, it was 85.76 and for TPOAb_GRS, it was 10.64.  Figure 2 shows the association of genetically determined 25 (OH) D with TPOAb concentration, and conversely, genetically determined TPOAb with 25 (OH) D concentrations. In the IV analysis, the causal regression coefficient of genetically determined 25 (OH) D for concentration of TPOAb was −0.720 (95% CI: −1.429, −0.012), and the causal regression coefficient of genetically determined TPOAb for 25 (OH) D was −0.087 (95% CI −0.271, 0.097).

## 4. Discussion

This investigation including 10636 community-dwelling Chinese adults, we examined whether these two GRSs, composed of SNPs significantly associated with genetically determined 25 (OH) D and genetically determined TPOAb concentration, were associated with increased TPOAb concentration and decreased 25 (OH) D level, respectively. Using the bidirectional MR study design, we found a causal role of vitamin D in the pathogenesis of increasing TPOAb level, while no causal relationship of higher TPOAb concentration to induce lower vitamin D status was found. To the best of our knowledge, for the first time, the results provided novel evidence for a causal relationship between genetically determined Vitamin D and increased TPOAb concentration by using MR. The identification of a causal relationship between 25 (OH) D and TPOAb concentration may have important clinical implications because vitamin D deficiency is common, and vitamin D supplementation is relatively safe and cost-effective [32].

The MR approach has an important benefit that it helps to overcome problems of confounding and reverse causality, which limits the ability to draw causal inferences in non-genetic observational studies [22]. However, several assumptions should be well met in performing a MR analysis [33–35]. First, the GRS as an IV was strongly associated with the exposure of interest. All SNPs used in this study have previously been shown to be significantly associated with vitamin D or TPOAb concentration in large meta-analysis of GWAS [29, 30]. In the present study, the associations of these two GRSs with the two corresponding exposures were also very significant. Second, the IVs must not be correlated with any confounders of the exposure-outcome association. In our study, we found the two GRSs were not associated with age, BMI, waist to hip ratio, and sex which were common potential confounders of the vitamin D-TPOAb association. We further tested the pleiotropic effects of each SNP on the above confounders and the results showed no SNP had pleiotropic effects. Third, the IV related to outcome only through the exposure of interest. There should be no direct effect of genotype on disease or any other mediated effectors other than through the exposure of interest. Thus, we analyzed the association of each SNP and GRSs with the corresponding outcome (vitamin D or TPOAb). All the SNPs and GRSs were not significantly associated with the outcome.

Recently, in a nationwide population-based study, data were obtained from the Korea National Health and Nutrition Examination Survey VI-1 and 2 (2013 and 2014) showed that the higher TPOAb level was more prevalent in the vitamin D-deficient group and vitamin D deficiency affects thyroid autoimmunity and dysfunction in iodine-replete area [36]. In our present study, we found that higher VD_GRS, presenting a low 25 (OH) D level, had a significantly negative association with higher TPOAb, providing strong evidence in support of a causal role of decreased vitamin D on increased TPOAb. These findings are consistent with evidence from observational studies that have demonstrated that low vitamin D levels influence risk of an increasing TPOAb level, and also in line with a very current case-control study which enrolled 200 euthyroid subjects: 100 newly diagnosed HT patients and 100 healthy individuals, matched for age, sex, and BMI, which aimed to investigate the association of HT with vitamin D status and SNPs of the vitamin D receptor (VDR). It first suggested that vitamin D deficiency may contribute to HT development and/or progression, acting as an environmental trigger [37].

Further, very limited intervention studies testing the effect of vitamin D supplementation on patients with AITD/HT showed that vitamin D supplements decreased TPOAb concentration, in those with vitamin D deficiency or those with normal vitamin D status [38, 39]. However, another randomized, double-blind, controlled trial (RCT) suggested Vitamin D_3_ supplementation did not affect the TPOAb level [40]. It should be noted that there exists an important difference between MR studies and RCTs. MR studies assess the association of a lifetime of exposure in the general population, whereas RCTs provide insights from supplementation for shorter periods in individuals at risk [41]. Thus, long-term RCTs may be needed to assess the role of vitamin D supplementation on the treatment of increased TPOAb adequately. The results from our study provides rationale to further investigate whether vitamin D supplementation may reduce AITDs susceptibility. The identification of vitamin D as a causal susceptibility factor for TPOAb may have important public health implications since vitamin D insufficiency/deficiency is common [11, 42], and vitamin D supplementation is both relatively safe and cost-effective [41].

The strength of our study included a relatively large sample size (more than 10 000 participants), well-defined community setting, and a highly homogeneous population. To our acknowledgment, this is the first report exploring the causal association between low vitamin D and high TPOAb concentration using the bidirectional MR study design, creating VD_GRS and TPOAb_GRS, representing the established common genetic variants of vitamin D and TPOAb level used as the IV.

However, several limitations should be acknowledged. First, it should be noted that the TPOAb_GRS we created in this study only for TPOAb concentration, not representing TPOAb positivity. Further studies were needed to explore the causal association between TPOAb positivity and vitamin D. Second, all participants were of were Han Chinese, a majority ethnic group indigenous within China (constitute about 92% of the population of the People's Republic of China). The findings of this study may not be generalizable other ethnicities. Third, 25 (OH) D was measured only once at baseline. Hence, we were not able to control intraindividual variability. Fourth, we build up our GRSs only based on common variants, which were considered to represent limited TPOAb and vitamin D heritability. We were unable to assess the potential contribution of rare variants.

## 5. Conclusion

We found that a higher VD_GRS was associated with higher risk of increased TPOAb concentration. This analysis provides evidence supporting a causal association between decreased vitamin D and increased concentration of TPOAb in an eastern Chinese population. Additional studies are needed to validate our findings and elucidate the mechanisms behind these findings.

## Figures and Tables

**Table 1:** Information of each SNP in GRS.

Gene	Chromosome	Position	SNP	Effect allele	EAF	*X* ^2^	*P* For HWE
Vitamin D-related SNPs							
GC	4	71742666	rs2282679	G	0.32	0.02	>0.05
CYP2R1	11	14893332	rs10741657	G	0.64	1.58	>0.05
DHCR7	11	71456403	rs12785878	G	0.55	1.04	>0.05
CYP24A1	20	54125940	rs6013897	A	0.16	7.61	0.005< *P* < 0.01
TPOAb-related SNPs							
HLA-DPB1	6	33087828	rs9277555	G	0.49	4.60	0.01< *P* < 0.05
TPO	2	1404043	rs11675434	T	0.31	0.94	>0.05
RERE	1	8429242	rs301799	C	0.17	0.28	>0.05
HCP5	6	31462150	rs3094228	G	0.15	7.31	0.005< *P* < 0.01

EAF, Effect allele frequency; GRS, gDQenetic risk score; HWE, Hardy-Weinberg equilibrium; SNP, Single nucleotide polymorphism.

**Table 2:** Association of each individual SNP with confounders.

SNP	Age		Waist to hip ratio		BMI		Sex	
	B (SE)	*P*	B (SE)	*P*	B (SE)	*P*	OR (95% CI)	*P*
Vitamin D-related SNPs								
rs2282679	0.185 (0.180)	0.306	0.000 (0.001)	0.916	0.023 (0.049)	0.638	0.983 (0.922, 1.049)	−0.017
rs10741657	−0.011 (0.177)	0.951	0.000 (0.001)	0.858	−0.017 (0.048)	0.728	1.023 (0.960, 1.089)	0.485
rs12785878	0.210 (0.171)	0.219	0.000 (0.001)	0.835	0.025 (0.046)	0.585	0.957 (0.901, 1.018)	0.162
rs6013897	0.185 (0.226)	0.412	−0.001 (0.001)	0.331	−0.037 (0.061)	0.548	0.935 (0.863, 1.013)	0.099
Vitamin D-related SNPs								
rs9277555	0.165 (0.171)	0.334	−0.001 (0.001)	0.322	0.030 (0.046)	0.521	1.007 (0.948, 1.070)	0.819
rs11675434	−0.120 (0.183)	0.512	0.002 (0.001)	0.083	0.031 (0.050)	0.529	1.014 (0.950, 1.083)	0.676
rs301799	−0.144 (0.224)	0.521	0.001 (0.001)	0.528	−0.007 (0.061)	0.909	1.033 (0.953, 1.119)	0.432
rs3094228	0.415 (0.236)	0.079	−0.001 (0.001)	0.684	0.054 (0.064)	0.398	1.046 (0.961, 1.138)	0.295

Data are expressed as unstandardized coefficients (standard error) and odds ratio (95% confidence interval). Multiple linear and logistic regression was performed. The model was adjusted for age (not for age), sex (not for sex), and waist to hip ratio (not for BMI and waist to hip ratio).

**Table 3:** Association of the GRSs with confounders.

	VD_GRS		TPOAb_GRS	
Continuous variable	B (SE)	*P*	B (SE)	*P*
Age	0.498 (0.346)	0.150	0.827 (1.123)	0.462
BMI	−0.016 (0.101)	0.871	0.422 (0.328)	0.199
Waist to hip ratio	0.000 (0.002)	0.808	0.003 (0.007)	0.625
Ln 25 (OH) D	−0.093 (0.009)	<0.001	−0.030 (0.031)	0.330
lnTPOAb	0.067 (0.033)	0.043	0.345 (0.107)	0.001
Categorical variable	OR (95%CI)	*P*	OR (95%CI)	*P*
Sex	0.931 (0.823, 1.054)	0.259	1.231 (0.823, 1.840)	0.311

Data are expressed as unstandardized coefficients (standard error) and odds ratio (95% confidence interval). Multiple linear and logistic regression was performed. The model was adjusted for age (not for age), sex (not for sex) and waist to hip ratio (not for BMI and waist to hip ratio).

**Table 4:** Characteristics of study participants according to the weighted vitamin D genetic risk score (VD_GRS) and weighted TPOAb_GRS (*n* = 10636).

Characteristics	VD_GRS			
	*Q*1	*Q*2	*Q*3	*P* For trend
VD_GRS	≤0.82	0.83 – 1.13	≥1.14	
N	3477	3623	3536	
Age, years	54.64 ± 13.02	54.76 ± 12.98	55.15 ± 12.69	0.219
Male (%)	39.2	40.7	40.0	0.439
Smokers (%)	19.1	20.9	20.6	0.146
BMI (kg/m^2^)	24.62 ± 3.49	24.63 ± 3.67	24.65 ± 3.59	0.941
Waist to hip ratio	0.86 ± 0.08	0.86 ± 0.08	0.86 ± 0.08	0.574
SBP (mmHg)	132.35 ± 21.24	132.55 ± 21.72	132.76 ± 21.69	0.738
25 (OH) D (mmol/L)	41.89 ± 13.22	40.80 ± 12.72	39.02 ± 11.96	<0.001
TPOAb (U/ml)	108.45 ± 289.42	113.84 ± 297.72	121.46 ± 310.48	0.019

	*TPOAb_GRS*			
	Q1	Q2	Q3	*P* For trend
TPOAb_GRS	≤0.136	0.137 – 0.240	≥0.241	
N	3660	3842	3134	
Age, years	54.72 ± 13.28	54.92 ± 12.68	54.92 ± 12.71	0.750
Male (%)	40.9	40.0	38.9	0.252
Smokers (%)	20.7	20.1	19.9	0.719
BMI (kg/m^2^)	24.54 ± 3.56	24.71 ± 3.60	24.64 ± 3.61	0.126
Waist to hip ratio	0.86 ± 0.08	0.86 ± 0.08	0.86 ± 0.08	0.242
SBP (mmHg)	132.67 ± 21.62	132.20 ± 21.41	132.87 ± 21.64	0.415
25 (OH) D (mmol/L)	40.59 ± 12.57	40.68 ± 12.81	40.40 ± 12.93	0.435
TPOAb (U/ml)	104.67 ± 283.87	119.49 ± 307.07	120.24 ± 307.21	0.021

The data are summarized as the mean ± SD for continuous variables or as a numerical proportion for categorical variables. *P* for trend was calculated by ANOVA and chi-square tests. 25 (OH) D, 25-hydroxyvitamin D; GRS, genetic risk score.

**Table 5:** Associations of VD_GRS and 25 (OH) D with TPOAb concentration.

	Model 1	Model 2
VD_GRS	0.066 (0.002, 0.129)	0.067 (0.002, 0.132)
Tertiles of VD_GRS		
* Q*1	0.000 (ref)	0.000 (ref)
* Q*2	0.009 (−0.045, 0.062)	0.007 (−0.047, 0.062)
* Q*3	0.061 (0.007, 0.114)	0.060 (0.006, 0.115)
*P* For trend	0.027	0.031
25 (OH) D	0.069 (0.003, 0.135)	0.070 (0.003, 0.137)

Data were presented as and 95% confidence interval. 25 (OH) D, 25-hydroxyvitamin D; GRS, genetic risk score; Model 1 adjusted for age and sex; Model 2 adjusted for terms in model 1 and waist to hip ratio.

**Table 6:** Associations of TPOAb_GRS and TPOAb concentration with 25 (OH) D.

	Model 1	Model 2
TPOAb_GRS	−0.031 (−0.090, 0.029)	−0.030 (−0.091, 0.030)
Tertiles of TPOAb_GRS		
* Q*1	0.000 (ref.)	0.000 (ref.)
* Q*2	0.003 (−0.012, 0.018)	0.003 (−0.012, 0.018)
* Q*3	−0.005 (−0.021, 0.011)	−0.005 (−0.021, 0.011)
*P* For trend	0.571	0.586
TPOAb	0.006 (0.000, 0.011)	0.006 (0.000, 0.011)

Data were presented as and 95% confidence interval. 25 (OH) D, 25-hydroxyvitamin D; GRS, genetic risk score; Model 1 adjusted for age and sex; Model 2 adjusted for terms in model 1 and waist to hip ratio.

## Data Availability

The data used to support the findings of this study are included within the article.

## Abbreviations

TPOAb: Thyroid peroxidase antibody
SPECT-China: Survey on prevalence in east China for metabolic diseases and risk factors
GRS: Genetic risk scores
MR: Mendelian randomization
HT: Hashimoto thyroiditis
25 (OH) D: 25-hydroxyvitamin D
IV: Instrumental variable.

## References

[1] Fommei E., Iervasi G. (2002). The role of thyroid hormone in blood pressure homeostasis: evidence from short-term hypothyroidism in humans. *Journal of Clinical Endocrinology & Metabolism*.

[2] Hajer G. R., van Haeften T. W., Visseren F. L. J. (2008). Adipose tissue dysfunction in obesity, diabetes, and vascular diseases. *European Heart Journal*.

[3] Duntas L. H., Brenta G. (2012). The effect of thyroid disorders on lipid levels and metabolism. *Medical Clinics of North America*.

[4] Du T., Yuan G., Zhang M., Zhou X., Sun X., Yu X. (2014). Clinical usefulness of lipid ratios, visceral adiposity indicators, and the triglycerides and glucose index as risk markers of insulin resistance. *Cardiovascular Diabetology*.

[5] Cogni G., Chiovato L. (2013). An overview of the pathogenesis of thyroid autoimmunity. *Hormones*.

[6] Choi Y. M., Kim W. G., Kim T. Y. (2014). Low levels of serum vitamin D3 are associated with autoimmune thyroid disease in pre-menopausal women. *Thyroid*.

[7] Vondra K., Stárka L., Hampl R. (2015). Vitamin D and thyroid diseases. *Physiological Research*.

[8] Shan Z., Chen L., Lian X. (2016). Iodine status and prevalence of thyroid disorders after introduction of mandatory universal salt iodization for 16 years in China: a cross-sectional study in 10 cities. *Thyroid*.

[9] Huber G., Staub J.-J., Meier C. (2002). Prospective study of the spontaneous course of subclinical hypothyroidism: prognostic value of thyrotropin, thyroid reserve, and thyroid antibodies. *Journal of Clinical Endocrinology & Metabolism*.

[10] Nielsen C. H., Brix T. H., Leslie R. G. Q., Hegedüs L. (2009). A role for autoantibodies in enhancement of pro-inflammatory cytokine responses to a self-antigen, thyroid peroxidase. *Clinical Immunology*.

[11] Palacios C., Gonzalez L. (2014). Is vitamin D deficiency a major global public health problem?. *The Journal of Steroid Biochemistry and Molecular Biology*.

[12] Baeke F., Takiishi T., Korf H., Gysemans C., Mathieu C. (2010). Vitamin D: modulator of the immune system. *Current Opinion in Pharmacology*.

[13] Kivity S., Agmon-Levin N., Zisappl M. (2011). vitamin D and autoimmune thyroid diseases. *Cellular and Molecular Immunology*.

[14] Tamer G., Arik S., Tamer I., Coksert D. (2011). Relative vitamin D insufficiency in Hashimoto’s thyroiditis. *Thyroid*.

[15] Mansournia N., Mansournia M. A., Saeedi S., Dehghan J. (2014). The association between serum 25OHD levels and hypothyroid Hashimoto’s thyroiditis. *Journal of Endocrinological Investigation*.

[16] Kim D. (2016). Low vitamin D status is associated with hypothyroid Hashimoto’s thyroiditis. *Hormones*.

[17] Muscogiuri G., Mari D., Prolo S. (2016). 25 hydroxyvitamin D deficiency and its relationship to autoimmune thyroid disease in the elderly. *International Journal of Environmental Research and Public Health*.

[18] Goswami R., Marwaha R. K., Gupta N. (2009). Prevalence of vitamin D deficiency and its relationship with thyroid autoimmunity in Asian Indians: a community-based survey. *British Journal of Nutrition*.

[19] Effraimidis G., Badenhoop K., Tijssen J. G. P., Wiersinga W. M. (2012). Vitamin D deficiency is not associated with early stages of thyroid autoimmunity. *European Journal of Endocrinology*.

[20] Yasmeh J., Farpour F., Rizzo V., Kheradnam S., Sachmechi I. (2016). Hashimoto thyroiditis not associated with vitamin D deficiency. *Endocrine Practice*.

[21] Jansen H., Samani N. J., Schunkert H. (2014). Mendelian randomization studies in coronary artery disease. *European Heart Journal*.

[22] Lawlor D. A., Harbord R. M., Sterne J. A. C., Timpson N., Davey Smith G. (2008). Mendelian randomization: using genes as instruments for making causal inferences in epidemiology. *Statistics in Medicine*.

[23] Davey Smith G., Ebrahim S. (2003). Mendelian randomization’: can genetic epidemiology contribute to understanding environmental determinants of disease?. *International Journal of Epidemiology*.

[24] Wang N., Chen Y., Ning Z. (2016). Exposure to famine in early life and nonalcoholic fatty liver disease in adulthood. *Journal of Clinical Endocrinology & Metabolism*.

[25] Chen Y., Chen Y., Wang N. (2017). Are thyroid nodules associated with sex-related hormones? A cross-sectional SPECT-China study. *BMJ Open*.

[26] Chen Y., Chen Y., Xia F. (2017). A higher ratio of estradiol to testosterone is associated with autoimmune thyroid disease in males. *Thyroid*.

[27] Wang N., Chen C., Zhao L. (2018). Vitamin D and nonalcoholic fatty liver disease: Bi-directional mendelian randomization analysis. *EBioMedicine*.

[28] Wang N., Cheng J., Ning Z. (2018). Type 2 diabetes and adiposity induce different lipid profile disorders: a mendelian randomization analysis. *The Journal of Cinical Endocrinology and Metabolism*.

[29] Schultheiss U. T., Teumer A., Medici M. (2015). A genetic risk score for thyroid peroxidase antibodies associates with clinical thyroid disease in community-based populations. *Journal of Clinical Endocrinology and Metabolism*.

[30] Li S.-S., Gao L.-H., Zhang X.-Y. (2016). Genetically low vitamin D levels, bone mineral density, and bone metabolism markers: a mendelian randomisation study. *Scientific Reports*.

[31] Vimaleswaran K. S., Berry D. J., Lu C. (2013). Causal relationship between obesity and vitamin D status: bi-directional Mendelian randomization analysis of multiple cohorts. *PLoS Medicine*.

[32] Ju S. Y., Jeong H. S., Kim D. H. (2014). Blood vitamin D status and metabolic syndrome in the general adult population: a dose-response meta-analysis. *Journal of Clinical Endocrinology and Metabolism*.

[33] Sheehan N. A., Didelez V., Burton P. R., Tobin M. D. (2008). Mendelian randomisation and causal inference in observational epidemiology. *PLoS Medicine*.

[34] Qi L. (2009). Mendelian randomization in nutritional epidemiology. *Nutrition Reviews*.

[35] Xu M., Bi Y., Huang Y. (2016). Type 2 diabetes, diabetes genetic score and risk of decreased renal function and albuminuria: a mendelian randomization study. *EBioMedicine*.

[36] Kim M., Song E., Oh H.-S. (2017). Vitamin D deficiency affects thyroid autoimmunity and dysfunction in iodine-replete area: Korea national health and nutrition examination survey. *Endocrine*.

[37] Giovinazzo S., Vicchio T. M., Certo R. (2017). Vitamin D receptor gene polymorphisms/haplotypes and serum 25 (OH) D3 levels in Hashimoto’s thyroiditis. *Endocrine*.

[38] Simsek Y., Cakır I., Yetmis M., Dizdar O. S., Baspinar O., Gokay F. (2016). Effects of Vitamin D treatment on thyroid autoimmunity. *Journal of Research in Medical Sciences: The Official Journal of Isfahan University of Medical Sciences*.

[39] Krysiak R., Szkróbka W., Okopień B. (2017). The effect of vitamin D on thyroid autoimmunity in levothyroxine-treated women with hashimoto’s thyroiditis and normal vitamin D status. *Experimental and Clinical Endocrinology & Diabetes: Official Journal, German Society of Endocrinology [and] German Diabetes Association*.

[40] Knutsen K. V., Madar A. A., Brekke M. (2017). Effect of vitamin D on thyroid autoimmunity: a randomized, double-blind, controlled trial among ethnic minorities. *Journal of the Endocrine Society*.

[41] Mokry L. E., Ross S., Ahmad O. S. (2015). Vitamin D and risk of multiple sclerosis: a mendelian randomization study. *PLoS Medicine*.

[42] Yetley E. A. (2008). Assessing the vitamin D status of the US population. *The American Journal of Clinical Nutrition*.

