# Erratum to “Bidirectional Mendelian Randomization Analysis for Vitamin D and Thyroid Peroxidase Antibody”

**DOI:** 10.1155/ije/9878270

**Published:** 2025-08-18

Y. Chen, B. Han, C. Zhu, et al., “Bidirectional Mendelian Randomization Analysis for Vitamin D and Thyroid Peroxidase Antibody,” *International Journal of Endocrinology* 2022 (2022): 2260388, https://doi.org/10.1155/2022/2260388.

In the article titled “Bidirectional Mendelian Randomization Analysis for Vitamin D and Thyroid Peroxidase Antibody,” there was an error in the Correspondence line. The corresponding author name “Yi Chen” was not included in the Correspondence line by error. The correct Correspondence line is shown below:

Correspondence should be addressed to Yi Chen; chenyi9h@126.com and Yingli Lu; luyingli2008@126.com

We apologize for this error.

